# Supplementary material for: Chromothripsis during telomere crisis is independent of NHEJ, and consistent with a replicative origin
Source: Genome Res. 2019 May;29(5):737–49. doi: 10.1101/gr.240705.118 (PMC6499312; doi:10.1101/gr.240705.118)
Supplement: Supplemental Material [file supp_gr.240705.118_Supplemental_file_1.zip › contigs/annotated_contigs/DB86/contig.2.DB86_length_739_mean_cov_6.48173207037.docx]

**DB86_length_739_mean_cov_6.48173207037**

```
TTTAAGTTCAAATTGCATTATTCTAAGAAGCTGTCACTTATGATAGACATTTAAGGCAACTTCCATTTAATTTGTTGATAATTTCTTTC
 >chr3:81745302-81745604 - E=7e-170
TTATGCCACAGGTAGCTTATCTGAGTATCGTTTTGCATTGTGGAGAGGTTTACAGAAATGTGGTAAATCTGTCAAAGCTGTACAGCATT

GTTTTTGCTCATCCAAGCAGTTGTTTCATGCAGGTGAATTGGAATTCACTGATTAGTCTTGACGCTTGGTCTGGTTTATTGTCAGGCTT

TGAATCTGTAGCTCTCTCTTCATTTCAGATTATT|T|AACTAGAAGGGAAGAATTGTAATGTTTCCAACACAAAGAAAAGGTAAATGTT
                                   >chr3:81874665-81875103 - E=1e-251
TGAGACAATTAATATTCCAATTACCCTGATTAGATTATTACACATTTTACATGTATACATGTAAAATATCACATGTATTCCCAAAACAT

GTACAACTATTATATATCAATTAAAAAAAACAAAGATAGTTTAGCATTTTTAAAGGAGGGGTACCAAAATTCAAACCCTATTATGTGAG

CTAAGTAACAATGAACATGTAAAAGGGCATGGAAAAGAAATATGAAAGATTAAAAGAAAGACTTCCATAGACACAGATATGCAGCATAG

ATTAGCTATTTTGTATTGTTCTTTGAAATATAAACTGAAATTCACTGCACTGTCATTTAGAGTTACTTTAATAAAAATATATTTGTTTC

TAATTTTTAGAAACTGGGCACTTGTGGTC
```
